# Widening Socioeconomic Inequalities in Smoking in Japan, 2001–2016

**DOI:** 10.2188/jea.JE20200025

**Published:** 2021-06-05

**Authors:** Hirokazu Tanaka, Johan P. Mackenbach, Yasuki Kobayashi

**Affiliations:** 1 Department of Public Health, Erasmus University Medical Center, Rotterdam, The Netherlands; 2 Department of Public Health, Graduate School of Medicine, the University of Tokyo, Tokyo, Japan

**Keywords:** epidemiology, smoking, socioeconomic factors, Japan, trends

## Abstract

**Background:**

Japan is one of the world’s largest tobacco epidemic countries but few studies have focused on socioeconomic inequalities. We aimed to examine whether socioeconomic inequalities in smoking have reduced in Japan in recent times.

**Methods:**

We analyzed data from the Comprehensive Survey of Living Conditions, a large nationally representative survey conducted every 3 years (*n* ≈ 700,000 per year) in Japan, during 2001–2016. Age-standardized smoking prevalence was computed based on occupational class and educational level. We calculated smoking prevalence difference (PD) and ratio (PR) of (a) manual workers versus upper non-manual workers and (b) low versus high educational level. The slope index of inequality (SII) and relative index inequality (RII) by educational level were used as inequality measures.

**Results:**

Overall smoking prevalence (25–64 years) decreased from 56.0% to 38.4% among men and from 17.0% to 13.0% among women during 2001–2016. The PD between manual and upper non-manual workers (25–64 years) increased from 11.9% (95% confidence interval [CI], 11.0–12.9%) to 14.6% (95% CI, 13.5–15.6%) during 2001–2016. In 2016, smoking prevalence (25–64 years) for low, middle, and highly educated individuals were 57.8%, 43.9%, and 27.8% for men, and 34.7%, 15.9%, and 5.6% for women, respectively. SII and RII by educational level increased among both sexes. Larger socioeconomic differences in smoking prevalence were observed in younger generations, which suggests that socioeconomic inequalities in smoking evolve in a cohort pattern.

**Conclusions:**

Socioeconomic inequalities in smoking widened between 2001 and 2016 in Japan, which indicates that health inequalities will continue to exist in near future.

## INTRODUCTION

Japan is the fourth largest tobacco-consuming country in the world. In 2010, the cigarette consumption in Japan was 1,904 cigarettes per person-year.^1^ Currently, preventing smoking-related diseases is an important public health concern because tobacco smoking remains a leading cause of death, especially among men.^2^ However, tobacco control in Japan has been evaluated to be insufficient in comparison to the World Health Organization (WHO) Framework Convention on Tobacco Control.^3^ Looking back to the long-term trends in the last half of the century, smoking prevalence decreased incredibly from 83.7% in 1966 to 29.7% in 2016 among men aged 20 years and over.^4^ However, smoking prevalence in Japan is higher than those of other high-income countries, such as the United States and several European countries.^5^ On the other hand, smoking prevalence among women in Japan is lower than in other high-income countries,^5^ while the decline of smoking prevalence among Japanese women has stagnated in recent years (see eFigure 1).^4^ Considering recent statistics on insufficient tobacco control policies, the tobacco epidemic is still an ongoing issue in Japan, and requires focused measures for a smoke-free society.

Socioeconomic inequalities in smoking are a key causal element of health inequalities.^6^ Socioeconomic inequalities in smoking persist in high-income countries, such as the United States, European countries, and South Korea.^7^^–^^10^ However, there is limited information regarding smoking prevalence by socioeconomic status in Japan due to lack of (1) a nation-wide surveillance system, (2) comprehensive evaluation of health behaviors, and (3) health policy for inequality reduction.^11^ Some Japanese studies revealed that income and occupation were clearly associated with the differences in smoking prevalence in different socioeconomic groups during the 2000s.^12^^,^^13^ Regarding educational inequalities, Tabuchi and Kondo reported that larger inequalities in smoking were found among young men and women in terms of both relative and absolute measures in 2010.^14^ However, we found no studies that addressed trends in socioeconomic inequalities in smoking in Japan.

Within this context, the aim of the present study is to examine the trends in smoking prevalence by socioeconomic status using a large nation-wide survey. We also assessed whether socioeconomic inequalities in smoking reduced in Japan with attention to a specific birth cohort.

## METHODS

### Data sources

We analyzed data from the Comprehensive Survey of Living Conditions (CSLC) conducted every 3 years from 2001 to 2016 (*n* ≈ 700,000 per year) in Japan, a large-scale national population-based survey by the Ministry of Health, Labour and Welfare (MHLW).^15^ Micro data was extracted and used with permission from MHLW. The CSLC was launched in 1986, and it has been conducted every year to collect nation-wide data about living conditions, such as family make-up, occupation, and income. The health status survey, an additional part of CSLC, has been conducted every 3 years from 1986, but the smoking status survey has been conducted only since 2001. According to MHLW, smoking prevalence (20 years and over) estimated by the CSLC were 48.4% for men and 14.0% for women in 2001, and 31.1% for men and 9.5% for women in 2016, respectively.^15^ This prevalence was similar to both the Japan Tobacco Inc. Japan Smoking Rate Survey (29.7% for men and 9.7% for women)^4^ in 2016 and the National Health and Nutrition Survey (30.2% for men and 8.2% for women) in 2016.^16^

Regarding the survey of 2016, the household questionnaire and health questionnaire covered whole households that were randomly sampled in 5,530 districts out of 1,040,000 districts (approximately 50 households in each district) defined in the 2015 National Census. Household questionnaire includes demographics (sex, age, marital status, family make-up, health insurance, pension, child rearing, working status, occupation, and educational background) whereas health questionnaire includes health status and medical help seeking behavior (subjective symptoms, regular visits to hospital/clinics, difficulties in daily life, self-assessed health status, mental health, health checkup, alcohol consumption, and smoking status). The sampling method is under consideration for improvement^17^; however, the CSLC is one of the most important survey in Japan. eTable 1 shows the survey participants’ demographic characteristics covering all survey years.

### Definition of smoking

Survey participants were asked whether they: (A) “never smoked,” (B) “smoked daily,” (C) “smoked occasionally but not every day,” or (D) “used to smoke daily (before at least 1 month)”, and the number of cigarettes consumed per day for current smokers. There was no distinction between smoking cigarettes, pipes, cigars, electronic cigarettes, and heat-not-burn tobacco products. Participants who answered (B) “smoked daily” or (C) “smoked occasionally but not every day”, were defined as ‘current smokers’.^14^

### Socioeconomic status

We used both occupational class and educational level to assess socioeconomic status because data for occupational class is available since 2001, whereas data for educational level was recorded only from 2010. Occupational class was divided into five categories: upper non-manual workers (eg, professionals, managers), lower non-manual workers (eg, clerical, service, sales workers), manual workers (eg, craft and related trades workers, semi-skilled and unskilled manual workers), farmers, and self-employed. This classification followed the Erikson-Goldthorpe-Portocarero (EGP) scheme.^18^ Economically inactive occupations/unknown categories were also identified according to participants’ responses. Educational level was classified into three categories: “low” (defined as International Standard Classification of Education [ISCED]: 1–2), “middle” (ISCED: 3–4), and “high” (ISCED: 5–6). In Japan, “low” corresponds to elementary school/junior high school graduates, “middle” corresponds to high school graduates and technical professional school graduates, and “high” corresponds to 2-year college, university, and graduate school.^19^ The detailed classifications are presented in eTable 2 and eTable 3. eTable 4 shows the distributions of educational level by occupational class estimated in the dataset.

### Analysis

The analyses were restricted to men and women aged 25–64 years old with regard to occupational class because retirement is common around the early 60s in Japan. As for educational level, we divided the sample into two age groups: 25–64 and 65–94 years old. We excluded survey participants aged 20–24 years old because this age band includes university and other higher education students. All analyses were conducted using weighted samples by sex. The weighting scores provided by MHLW were recalibrated to obtain average weight for all study participants equal to one, which resulted in the standard errors being approximated to those of the unweighted sample when calculating age-standardized smoking prevalence.

First, we calculated crude smoking prevalence by occupational class, educational level, sex, and 5-year age groups (20–94 years old) to assess age-specific smoking prevalence. Birth cohort-specific smoking prevalence differences between manual workers and upper non-manual workers were calculated to assess changes within the birth cohort between 2001 and 2016.

Second, age-standardized smoking prevalence by occupational class and educational level was computed using the data in 5-year age intervals. The 2013 European standard population was used as reference for direct standardization because (1) the distribution is similar to what was observed in the 2000 Japanese Census (see eFigure 2); (2) the 1985 Japanese standard population, which is generally used for standardization for official Japanese statistics, deviates from the recent Japanese population distribution; and (3) the recent Japanese standard population (eg, in 2010) has not yet been officially established. The changes in smoking prevalence during the study period were simply calculated by subtraction of data values at two points between 2001 and 2016 (for occupational class) or 2010 and 2016 (for educational level).

Finally, we calculated smoking prevalence difference (PD) and prevalence ratio (PR) of manual versus upper non-manual workers and low versus high educational level to measure inequalities. A bootstrap procedure with 1,000 replications was used to calculate 95% confidence intervals (CIs). We calculated the Average Inter-group Differences (AID) as inequality measures for occupational class because not all strata of occupational class can be hierarchically ordered.^20^^,^^21^ The slope index of inequality (SII) and relative index inequality (RII) were used as inequality measures for educational level.^20^^,^^21^ Both SII and RII were calculated adjusting for 5-year age groups.

## RESULTS

### Smoking prevalence differences by age (birth cohort)

**Figure 1.**
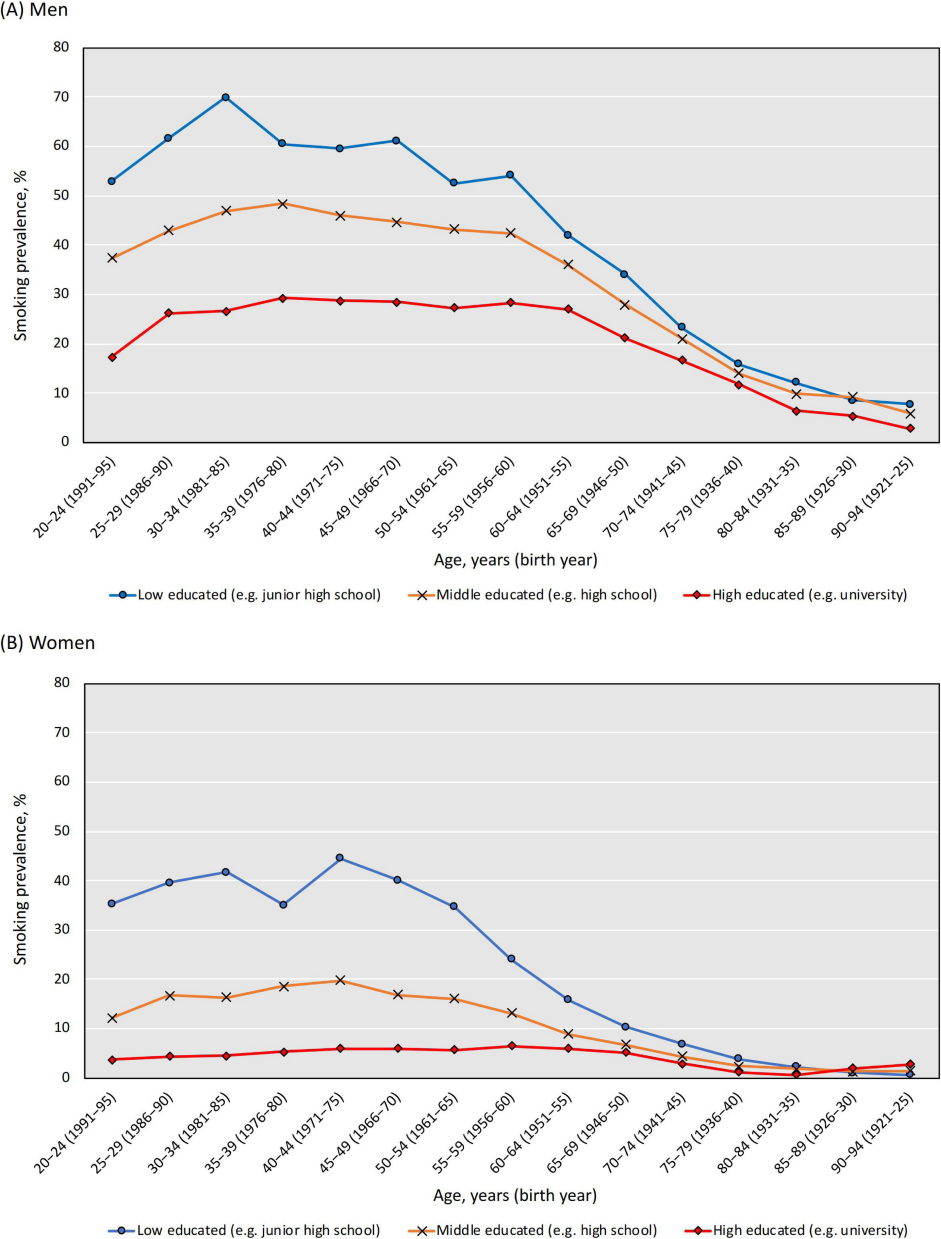
Smoking prevalence by educational level and age (birth year) in Japan in 2016

Figure 1 shows crude smoking prevalence by educational level and age in 2016 (including 20–24 years old). Substantial differences were observed among working-age (20–64 years old) but the differences were not substantial among 65 years old and over for both sexes. Clear differences in smoking prevalence by occupational class were also observed (presented in eFigure 3).

Table 1 shows changes in birth cohort-specific PD by occupational class between 2001 and 2016. We found that the inequalities within the birth cohort were carried over through the period (eg, PD [15.7%] between male manual and upper non-manual workers who were born between 1966–1970 [aged 30–34 in 2001] to PD [14.7%] in 2016). The similar trend was observed among women (eg, PD [14.1%] between female manual and upper non-manual workers who were born between 1966–1970 [aged 30–34 in 2001] to PD [10.6%] in 2016). Therefore, the substantial socioeconomic inequalities in smoking were mainly prevalent in the working-aged (25–64 years old) men and women during the period of our study.

**Table 1.** Changes in the birth cohort-specific smoking prevalence difference (PD) by occupational class between 2001 and 2016

Birth year	Men				Women			
	2001		2016		2001		2016	
	Age	PD^*^ (%)	Age	PD^*^ (%)	Age	PD^*^ (%)	Age	PD^*^ (%)
1986–1990	—	—	25–29	16.1	—	—	25–29	11.0
1981–1985	—	—	30–34	16.0	—	—	30–34	12.8
1976–1980	—	—	35–39	16.9	—	—	35–39	9.9
1971–1975	25–29	12.9	40–44	14.6	25–29	10.1	40–44	10.9
1966–1970	30–34	15.7	45–49	14.7	30–34	14.1	45–49	10.6
1961–1965	35–39	13.9	50–54	12.3	35–39	5.7	50–54	4.1
1956–1960	40–44	14.3	55–59	14.0	40–44	1.6	55–59	3.7
1951–1955	45–49	9.8	60–64	12.1	45–49	2.9	60–64	0.1
1946–1950	50–54	9.7	—	—	50–54	−0.7	—	—
1941–1945	55–59	12.5	—	—	55–59	2.5	—	—
1936–1940	60–64	6.3	—	—	60–64	−3.0	—	—
(Age-standardized PD)^**^		(11.9)		(14.6)		(4.1)		(8.0)

^*^Prevalence difference (PD) was calculated as PD = prevalence_(manual workers)_ − prevalence_(upper non-manual workers)_.

^**^Age-standardized prevalence difference (PD) among men and women aged 25–64 (the same results shown in Table 3). Survey participants aged 20–24 years old were excluded because this age band includes university and other higher education students.

### Trends in smoking prevalence by occupational class and educational level

Table 2 shows the changes in age-standardized smoking prevalence by occupational class and educational level; smoking prevalence every 3 years is presented in eTable 5. Smoking prevalence decreased from 56.0% (95% CI, 55.8–56.3%) to 38.4% (95% CI, 38.1–38.6%) among men aged 25–64 and from 17.0% (95% CI, 16.8–17.2%) to 13.0% (95% CI, 12.8–13.1%) among women aged 25–64 during the period. In 2001, manual workers had the highest smoking prevalence (61.2%) among men, whereas self-employed individuals had the highest smoking prevalence (27.2%) among women. The trends remained among men, but manual workers had the highest smoking prevalence (18.7%) among women in 2016. Smoking prevalence for ‘low’, ‘middle’, and ‘high’ educated men aged 25–64 years old were 57.8% (95% CI, 56.6–59.0%), 43.9% (95% CI, 43.5–44.4%), and 27.8% (95% CI, 27.4–28.2%), respectively. Smoking prevalence for ‘low’, ‘middle’, and ‘high’ educated women aged 25–64 years old were 34.7% (95% CI, 33.3–36.1%), 15.9% (95% CI, 15.6–16.2%), and 5.6% (95% CI, 5.4–5.8%), respectively. Among the elderly, smoking prevalence for low, middle, and highly educated men were 21.6% (95% CI, 20.9–22.2%), 18.5% (95% CI, 18.1–19.0%), 14.1% (95% CI, 13.5–14.7%), and 5.9% (95% CI, 5.6–6.3%), 4.0% (95% CI, 3.8–4.2%), and 2.9% (95% CI, 2.5–3.2%) for women, respectively.

**Table 2.** Trends in age-standardized smoking prevalence by occupational class and educational level

Survey year	2001		2010		2016		Change		
	%	95% CI	%	95% CI	%	95% CI	%	Percent change^*^ %	Annual prevalence change^**^ %
Men							(2001–2016)		
All population (aged 25–94)^***^	49.3	(49.1–49.6)	37.5	(37.3–37.7)	33.1	(32.9–33.3)	−16.3	−33.0	−1.0
All population (aged 25–64)	56.0	(55.8–56.3)	43.7	(43.4–44.0)	38.4	(38.1–38.6)	−17.7	−31.5	−1.1
All population (aged 65–94)	31.0	(30.5–31.4)	20.4	(20.0–20.8)	18.5	(18.2–18.8)	−12.4	−40.1	−0.8
Occupational class (EGP scheme, aged 25–64)							(2001–2016)		
Upper non-manual workers (I+II)	50.0	(49.4–50.5)	37.5	(37.1–38.0)	32.5	(32.1–33.0)	−17.4	−34.9	−1.1
Lower non-manual workers (III)	55.6	(55.0–56.1)	42.9	(42.3–43.4)	37.2	(36.6–37.7)	−18.4	−33.1	−1.2
Manual workers (V+VI+VIIa)	61.9	(61.4–62.5)	53.3	(52.7–53.9)	47.1	(46.5–47.7)	−14.8	−23.9	−0.9
Farmers (IVc+VIIb)	58.1	(56.4–59.7)	49.4	(47.4–51.5)	45.8	(43.7–47.8)	−12.3	−21.2	−0.8
Self-employed (IVa+b)	61.2	(60.4–62.0)	48.1	(47.1–49.2)	45.3	(44.0–46.5)	−16.0	−26.0	−1.0
Economically inactive/unknown	54.7	(53.9–55.5)	44.4	(43.6–45.2)	38.3	(37.5–39.0)	−16.5	−30.1	−1.0
Educational level (aged 25–64)							(2010–2016)		
Low (ISCED: 1, 2)	N/A		60.0	(58.9–61.0)	57.8	(56.6–59.0)	−2.2	−3.6	−0.4
Middle (ISCED: 3, 4)			49.2	(48.8–49.6)	43.9	(43.5–44.4)	−5.3	−10.7	−0.9
High (ISCED: 5, 6)			32.9	(32.5–33.4)	27.8	(27.4–28.2)	−5.2	−15.7	−0.9
Educational level (aged 65–94)							(2010–2016)		
Low (ISCED: 1, 2)	N/A		22.8	(22.1–23.5)	21.6	(20.9–22.2)	−1.2	−5.3	−0.2
Middle (ISCED: 3, 4)			20.5	(19.9–21.1)	18.5	(18.1–19.0)	−2.0	−9.8	−0.3
High (ISCED: 5, 6)			15.2	(14.4–16.0)	14.1	(13.5–14.7)	−1.1	−7.1	−0.2
Women							(2001–2016)		
All population (aged 25–94)^***^	14.1	(14.0–14.3)	12.1	(12.0–12.2)	10.7	(10.6–10.9)	−3.4	−24.0	−0.2
All population (aged 25–64)	17.0	(16.8–17.2)	14.9	(14.7–15.1)	13.0	(12.8–13.1)	−4.1	−23.9	−0.3
All population (aged 65–94)	6.1	(5.8–6.3)	4.5	(4.3–4.6)	4.5	(4.4–4.7)	−1.5	−24.8	−0.1
Occupational class (EGP scheme, aged 25–64)							(2001–2016)		
Upper non-manual workers (I+II)	14.7	(14.1–15.3)	12.2	(11.8–12.7)	10.8	(10.4–11.2)	−4.0	−26.9	−0.2
Lower non-manual workers (III)	18.6	(18.2–19.0)	16.1	(15.8–16.5)	13.8	(13.5–14.1)	−4.8	−25.9	−0.3
Manual workers (V+VI+VIIa)	18.8	(18.1–19.5)	19.6	(18.7–20.5)	18.7	(17.9–19.6)	−0.1	−0.5	0.0
Farmers (IVc+VIIb)	14.2	(12.7–15.7)	10.4	(8.3–12.4)	11.5	(9.2–13.7)	−2.8	−19.5	−0.2
Self-employed (IVa+b)	27.2	(25.8–28.7)	20.8	(19.3–22.2)	16.7	(15.1–18.3)	−10.5	−38.6	−0.7
Economically inactive/unknown	15.9	(15.6–16.2)	14.0	(13.7–14.3)	12.2	(11.9–12.5)	−3.8	−23.6	−0.2
Educational level (aged 25–64)							(2010–2016)		
Low (ISCED: 1, 2)	N/A		37.9	(36.5–39.2)	34.7	(33.3–36.1)	−3.1	−8.3	−0.5
Middle (ISCED: 3, 4)			17.5	(17.2–17.8)	15.9	(15.6–16.2)	−1.6	−9.0	−0.3
High (ISCED: 5, 6)			7.4	(7.2–7.7)	5.6	(5.4–5.8)	−1.8	−24.9	−0.3
Educational level (aged 65–94)							(2010–2016)		
Low (ISCED: 1, 2)	N/A		5.4	(5.1–5.8)	5.9	(5.6–6.3)	0.5	9.2	0.1
Middle (ISCED: 3, 4)			3.8	(3.5–4.0)	4.0	(3.8–4.2)	0.2	5.3	0.0
High (ISCED: 5, 6)			2.7	(2.1–3.2)	2.9	(2.5–3.2)	0.2	7.0	0.0

^*^Difference percentages expressed as percentages of 2001 (for occupational class) or 2010 (for educational level).

^**^Estimated from prevalence of two points between 2001–2016 (for occupational class) or 2010–2016 (for educational level).

^***^Survey participants aged 20–24 years old were excluded because this age band includes university and other higher education students.

CI, confidence interval.

EGP scheme: Erikson-Goldthorpe-Portocarero scheme, ISCED: International Standard Classification of Education.

Low (ISCED: 1, 2): elementary school/junior high school graduation.

Middle (ISCED: 3, 4): high school/technical professional school graduation.

High (ISCED: 5, 6): 2-year college/university graduation and more.

We found smoking prevalence reduction pace more prominent among upper non-manual workers (−1.1% per year for men) than those of manual workers (−0.9% per year for men) for both sexes between 2001 and 2016. The similar pattern was also found for educational inequalities (annual reduction pace: −0.9% per year among high educated versus −0.4% among low educated for men between 2010 and 2016).

### Changes in inequalities measures

Table 3 shows changes in inequality measures of smoking prevalence by occupational class and educational level. The PD between manual workers and upper non-manual workers increased from 11.9% (95% CI, 11.0–12.9%) in 2001 to 14.6% (95% CI, 13.5–15.6%) in 2016 and the PR also increased from 1.24 (95% CI, 1.22–1.26) in 2001 to 1.45 (95% CI, 1.41–1.49) in 2016 among men aged 25–64 years old. For women, the PD between manual workers and upper non-manual workers increased from 4.1% (95% CI, 2.9–5.2%) in 2001 to 7.9% (95% CI, 6.8–9.1%) in 2016 and the PR also increased from 1.28 (95% CI, 1.19–1.37) in 2001 to 1.74 (95% CI, 1.61–1.87) in 2016. AIDs also indicated that inequality increased by occupational class: AID (relative version: Gini coefficient like measure) increased from 4.7% in 2001 to 8.5% in 2016 for men. A similar trend was observed among working-age women: AID (relative version) increased from 7.1% to 8.8% between 2001 and 2016.

**Table 3.** Trends in inequalities measures of smoking prevalence by occupational class and educational level

Survey year	2001		2010		2016	
	Point estimates	95% CI	Point estimates	95% CI	Point estimates	95% CI
Men						
Occupational class						
Prevalence difference (PD)^a^ (%)	11.9	(11.0–12.9)	15.8	(14.7–16.8)	14.6	(13.5–15.6)
Prevalence ratio (PR)^b^	1.24	(1.22–1.26)	1.42	(1.39–1.45)	1.45	(1.41–1.49)
Average Inter-group Difference ​ (AID absolute version)^*^	2.6		3.4		3.3	
Average Inter-group Difference ​ (AID relative version) (%)^*^	4.7		7.7		8.5	
Educational level (aged 25–64)^**^						
Prevalence difference (PD)^c^ (%)	N/A		27.0	(25.6–28.5)	30.0	(28.4–31.7)
Prevalence ratio (PR)^d^			1.79	(1.74–1.85)	2.05	(1.98–2.12)
Slope Index of Inequality (SII) (%)			46.7	(44.7–48.8)	51.8	(49.7–53.9)
Relative Index of Inequality (RII)			2.26	(2.18–2.35)	2.83	(2.72–2.96)
Educational level (aged 65–94)						
Prevalence difference (PD)^c^ (%)	N/A		7.6	(6.1–9.1)	7.5	(6.3–8.6)
Prevalence ratio (PR)^d^			1.50	(1.37–1.63)	1.53	(1.42–1.64)
Slope Index of Inequality (SII) (%)			8.8	(7.2–10.4)	9.7	(8.3–11.1)
Relative Index of Inequality (RII)			1.50	(1.39–1.62)	1.64	(1.53–1.75)
Women						
Occupational class						
Prevalence difference (PD)^a^ (%)	4.1	(2.9–5.2)	7.4	(6.1–8.7)	7.9	(6.8–9.1)
Prevalence ratio (PR)^b^	1.28	(1.19–1.37)	1.60	(1.48–1.73)	1.74	(1.61–1.87)
Average Inter-group Difference ​ (AID absolute version)^*^	1.2		1.3		1.1	
Average Inter-group Difference ​ (AID relative version) (%)^*^	7.1		8.8		8.8	
Educational level (aged 25–64)^**^						
Prevalence difference (PD)^c^ (%)	N/A		30.5	(28.6–32.3)	29.1	(27.2–31.1)
Prevalence ratio (PR)^d^			5.00	(4.63–5.37)	5.97	(5.49–6.45)
Slope Index of Inequality (SII) (%)			36.2	(35.0–37.4)	37.5	(36.3–38.7)
Relative Index of Inequality (RII)			7.56	(7.09–8.06)	10.1	(9.44–10.9)
Educational level (aged 65–94)						
Prevalence difference (PD)^c^ (%)	N/A		2.8	(1.8–3.7)	3.1	(2.3–3.9)
Prevalence ratio (PR)^d^			2.04	(1.33–2.75)	2.08	(1.58–2.58)
Slope Index of Inequality (SII) (%)			3.5	(2.8–4.2)	3.9	(3.2–4.5)
Relative Index of Inequality (RII)			2.19	(1.85–2.58)	2.30	(2.00–2.65)

^a^Prevalence difference (PD) was calculated as PD = prevalence_(manual workers)_ − prevalence_(upper non-manual workers)_ with age-standardized.

^b^Prevalence ratio (PR) was calculated as PR = prevalence_(manual workers)_/prevalence_(upper non-manual workers)_ with Poisson regression model controlling age-category.

^c^Prevalence difference (PD) was calculated as PD = prevalence_(low)_ − prevalence_(high)_ with age-standardized.

^d^Prevalence ratio (PR) was estimated as PR = prevalence_(low)_/prevalence_(high)_ with Poisson regression model controlling age-category.

^*^Regarding the Average Inter-group Difference (AID), we calculated the point estimates only.

^**^Survey participants aged 20–24 years old were excluded because this age band includes university and other higher education students.

CI, confidence interval.

Educational inequalities in smoking were more prominent than that of occupational class. The PD between low and high educated was 30.0% (95% CI, 28.4–31.7%) and the PR was 2.05 (95% CI, 1.98–2.12) among men aged 25–64 years old in 2016. For women, the PD between low and high educated was 29.1% (95% CI, 27.2–31.1%) and the PR was 5.97 (95% CI, 5.49–6.45) among women aged 25–64 years old in 2016. Both SII and RII by educational level increased among both men and women: RII increased from 2.26 (95% CI, 2.18–2.35) in 2010 to 2.83 (95% CI, 2.72–2.96) in 2016 among men aged 25–64 years old. SII slightly increased from 36.2 (95% CI, 35.0–37.4) to 37.5 (95% CI, 36.3–38.7) among women aged 25–64 years old between 2010 and 2016.

## DISCUSSION

### Strengths and limitations

This is the first study that assessed whether socioeconomic inequalities in smoking had reduced in recent years in Japan using a large nationally representative survey. The results indicated that socioeconomic inequalities in smoking widened between 2001 and 2016 in Japan while overall smoking prevalence was decreasing. Our findings shed more light on useful benchmarks and entry points for reducing the incidence of smoking-related diseases in specific populations and eliminating health inequalities.

Smoking status and socioeconomic status were self-reported as a part of health survey of the CSLC; this is a potential limitation of our study. Smoking status response rates were high (90–95%), but those of occupational level and educational level were relatively low (85–90%). This may distort the estimation of smoking prevalence by socioeconomic status. However, smoking prevalence estimated by the CSLC was close to those of other national-level surveys.^4^^,^^16^

### Interpretation

Socioeconomic inequalities in smoking are associated with inequalities in mortality from smoking-related causes, such as lung cancer and ischemic heart disease. The contributions of smoking to socioeconomic inequalities in mortality were 19–55% among men and −1–56% among women in European countries.^6^ We are deeply concerned about widening inequalities in smoking-attributable mortality in Japan because our findings suggested that socioeconomic differences among working-age men and women (approximately born between 1951 and 1990) mainly contributed to these inequalities. A trend analysis, reported by Funatogawa et al, showed that smoking prevalence at the age of 20–29 was very high (about 80%) among men who were born between 1925 and 1955.^22^ This implies that socioeconomic inequalities had already existed among the birth cohort since our results confirmed the existence of socioeconomic difference in smoking among men who were born after 1921 (shown in Figure 1 and Table 1). The same study also revealed that the start of increase in smoking prevalence was observed among women who were born in 1965 (smoking prevalence: 18% at the age of 20–29 years) and smoking prevalence peaked among women who were born in 1975 (23% at the age of 20–29 years).^22^ We interpret that this increase was affected by the promotion of women’s participation in the workforce; the Equal Employment Opportunity Law was implemented in 1986 in Japan. Therefore, our findings complemented that absolute socioeconomic inequalities especially widened among women who were born between 1966 and 1990 as smoking habits peaked up.

Current socioeconomic differences in smoking among working-age people who were born between 1951 and 1990 (aged 25–64 years in 2016) are likely to contribute to socioeconomic inequalities in mortality in near future. According to the Japanese vital statistics in 2015, around 93.0% of deaths in the Japanese population occurred at the age of 60 years and over.^23^ Hence, the substantial socioeconomic differences in smoking will be carried over and directly contribute to socioeconomic inequality in mortality (eg, men and women who were born between 1951 and 1990 turn 60–99 years old in 2050), taking the lag period between smoking and smoking-attributable disease onset into consideration. Our findings emphasis socioeconomic inequalities in smoking are very likely to widen health inequalities although health inequalities in Japan were reported to be less than those of Western countries.^24^ The estimated smoking-related mortality was similar by occupational class in 2015 (32 and 30 per 100,000 person-years for upper non-manual workers and manual workers among men aged 35–64 years, respectively)^25^ but mortality difference may widen owning to the widening of smoking prevalence difference by occupational class as confirmed by our study.

In Japan, the number of smokers has been decreasing over the decades.^4^ Our findings suggest that this is partly because of the increasing the level of educational achievement. The percentages of individuals who were ‘low’ educated (eg, junior high school graduation) were 5.9% for men and 4.3% for women aged 25–64 years in 2016 whereas the percentages were 27.8% for men and 33.9% for women aged 65–94 years (eTable 1). Increasing level of educational achievement may promote reduction of smoking prevalence by avoiding the social environment that is tolerant toward initiation of smoking, since less educational attachment is strongly associated with a high possibility of developing the smoking habit.

### International comparisons

Not many studies have focused on an international comparison of socioeconomic inequalities in smoking between Japan and Western countries. A study reported that inequalities by occupational class in smoking prevalence among Japanese civil servants were smaller than in Britain and Finland.^26^ However, our findings confirmed that socioeconomic inequalities are remarkable at the national level, which suggests that socioeconomic inequalities in smoking are not smaller than other high-income countries.^7^^–^^10^ Moreover, the patterns of widening socioeconomic inequalities, smaller smoking prevalence reduction pace among disadvantaged groups (shown in Table 2), were suggested to be comparable with those observed in the United States.^8^

Smoking prevalence was comparable with those observed in South Korea: smoking prevalence of non-manual and manual workers were 35.0% and 45.8% among men in 2016, respectively.^10^ In Japan and South Korea, however, the patterns of mortality inequalities by occupational class from smoking-related causes were different from those in European countries; manual workers had lower mortality than upper non-manual workers.^25^ Further analysis is needed to assess these discrepancies.

### Policy implications

We need to consider three aspects to get the overall picture regarding changes in health inequalities; (1) the trend in the average prevalence of smoking, (2) the trend in absolute inequalities, and (3) the trend in relative inequalities.^27^ Our study revealed that the average prevalence of smoking decreased, trends in absolute inequalities in smoking were stable, and relative inequalities in smoking increased. Apparently, Japan has insufficiently addressed socioeconomic inequalities in smoking. In Europe, pleas have been made for equity-oriented tobacco control policies which are based on statistics about socioeconomic inequalities in smoking and which include measures that have been shown to reduce inequalities in smoking, particularly, raising the price of cigarettes.^28^ In Japan, such policies are still lacking, and the average retail price of cigarettes is cheaper than that in other countries.^29^ Policymakers in Japan should consider raising the price of cigarettes in order to reduce socioeconomic inequalities in smoking.

In Japan, the situation surrounding tobacco policy is unique in terms of tobacco taxation, smoke-free legislation, measures to reduce socioeconomic inequalities including anti-tobacco campaigns.^11^^,^^29^ Japan ratified the WHO Framework Convention on Tobacco Control in 2004 but the implementation of tobacco control is not fulfilled sufficiently.^3^ For example, MHLW tried to introduce a smoking ban in public indoor spaces to prevent secondhand smoke exposure in 2017; however, the suggested policy was intensely opposed by pro-tobacco policymakers.^30^ Moreover, Japanese policymakers are generally reluctant to take tobacco control measures, just like their counterparts in other East-Asian countries (eg, South Korea and China),^29^ because tobacco products largely contribute to national income. Our findings, however, suggest that such a fiscal-oriented policy would not only delay tobacco control but also exacerbate the situations of health inequalities caused by smoking. Policymakers should not focus on the contributions of tobacco to tax but should regard tobacco-free policy as important for achieving equity in the society.

There is another issue that cannot be overlooked in the Japanese work environment: more workers are recently prohibited to smoke in their workplace. For example, the smoking area is being closed at many locations (eg, offices, city halls, stations, and schools).^31^ Moreover, several firms recently announced that they refrain from adopting smokers based on their new recruit policy.^32^ Within this context, there is a serious concern that the ongoing policies regarding smoking habits are being propagated without considering socioeconomic differences in smoking and evidence-based measures for reducing socioeconomic inequalities. Taking our findings into considerations, policymakers should be aware of the strong relationship between socioeconomic status and smoking prevalence and of the underlying causes of such a relationship, and should promote policies to support cessation of smoking among groups with high smoking prevalence (eg, manual workers and low educated). Further investigation into the fairness of the labor market with regard to the smoking habit is necessary.

Indeed, a nation-wide surveillance system is necessary to monitor changes in socioeconomic inequalities in smoking in Japan.^11^ For the time being, the CSLC plays an important role in the nation-wide survey regarding smoking. Moreover, nation-wide surveillance should include the situations of second-hand smoke exposure,^11^^,^^33^ as well as smoking by socioeconomic status, and new smoking methods (eg, heat-not-burn tobacco).^34^

### Conclusions

Socioeconomic inequalities in smoking widened between 2001 and 2016 in Japan, while smoking prevalence was decreasing. Our findings showed that socioeconomic differences in smoking among a specific birth cohort (approximately born between 1951 and 1990; working-age men and women in 2016) mainly contributed to inequalities. This suggests that the tobacco epidemic still strongly runs over generations and health inequalities are likely to continue across generations as well. We emphasis reducing socioeconomic inequalities in smoking critically depends on focusing on reducing smoking prevalence among manual workers and low/middle educated men and women. Equity-oriented tobacco control policies for eliminating socioeconomic inequalities in smoking are strongly required; otherwise, smoking will remain a potential factor responsible for health inequality due to smoking-related diseases.

## APPENDIX A. SUPPLEMENTARY DATA

eTable 1. Number of survey participants

eTable 2. Definitions of occupational class

eTable 3. Definitions of educational level

eTable 4. Distributions of educational level by occupational class (EGP scheme, aged 25–64)

eTable 5. Trends in age-standardized smoking prevalence rates by occupational class and educational level

eFigure 1. Trends in smoking prevalence reported by Japan Tobacco Inc. Japan Smoking Rate Survey, 1965–2018 (data source: http://www.health-net.or.jp/tobacco/product/pd090000.html)

eFigure 2. Comparison of distribution between the Japanese population in 2000 and the 2013 European Standard Population

eFigure 3. Smoking prevalence by occupational class and age (birth year) in Japan in 2016
