# Supplementary material for: Widening Socioeconomic Inequalities in Smoking in Japan, 2001–2016
Source: J Epidemiol. 2021 Jun 5;31(6):369–77. doi: 10.2188/jea.JE20200025 (PMC8126678; doi:10.2188/jea.JE20200025)
Supplement: Supplementary file 1 [file je-31-369-s001.pdf]

Supplementary file

Tanaka H, Mackenbach JP, Kobayashi Y. Widening socioeconomic inequalities in smoking in Japan, 2001-2016

Contents:

eFigure 1. Trends in smoking prevalence reported by Japan Tobacco Inc. Japan Smoking Rate Survey, 1965-2018 (page 2)

eTable 1. Number of survey participants (page 3-4)

eTable 2. Definitions of occupational class (page 5)

eTable 3. Definitions of educational level (page 6)

eTable 4. Distributions of educational level by occupational class (page 7)

eFigure 2. Comparison of distribution between the Japanese population in 2000 and the 2013 European Standard Population (page 8)

eFigure 3. Smoking prevalence by occupational class and age (birth year) in Japan in 2016 (page 9)

eTable 5. Trends in age-standardized smoking prevalence rates by occupational class and educational level (page 10-12)

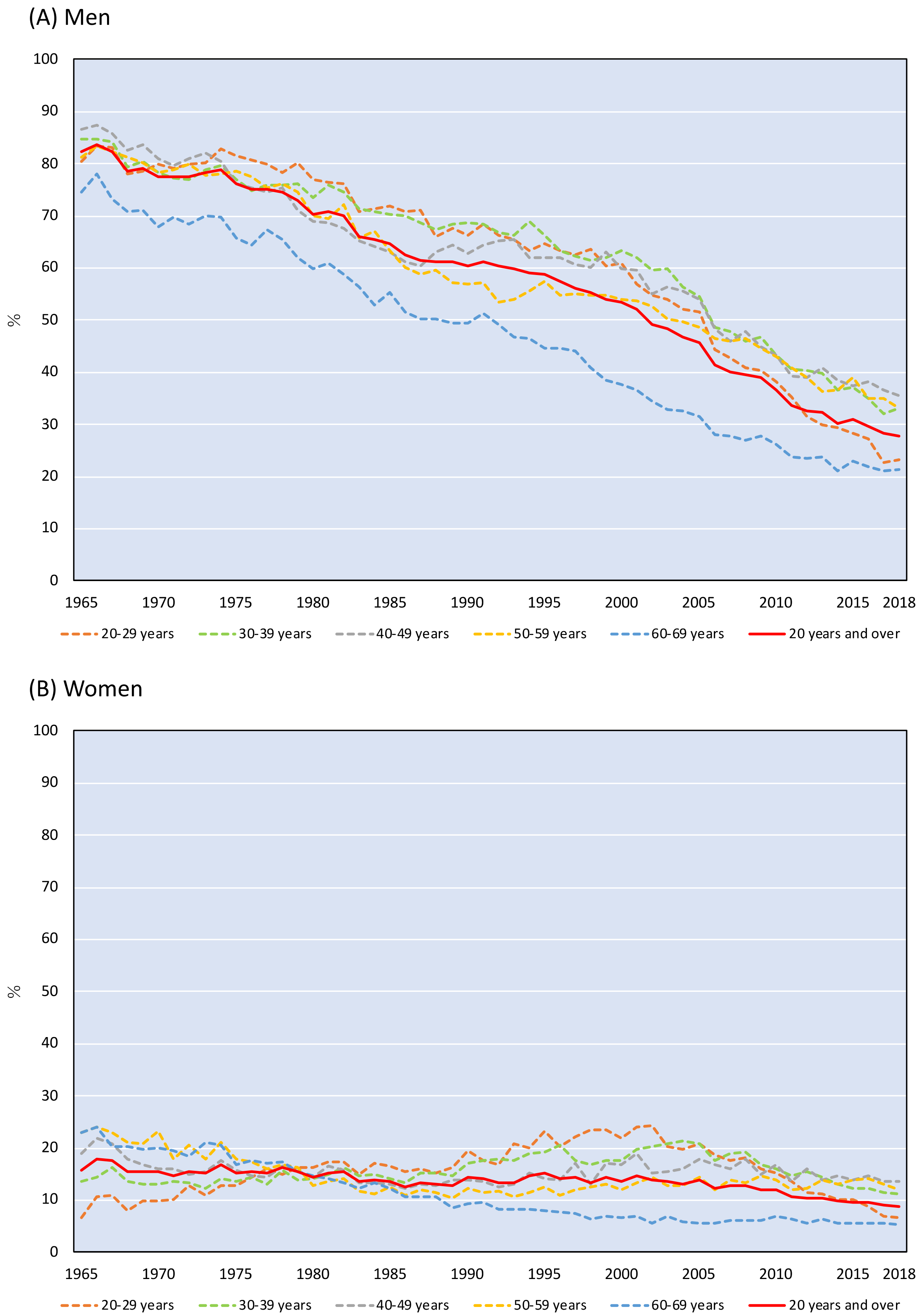

eFigure 1. Trends in smoking prevalence reported by Japan Tobacco Inc. Japan Smoking Rate Survey, 1965–2018 (data source: http://www.health-net.or.jp/tobacco/product/pd090000.html)

eTable 1. Number of survey participants

Survey year	2001			2004			2007			2010			2013			2016		
	n	(%)	Weighted (%)	n	(%)	Weighted (%)	n	(%)	Weighted (%)	n	(%)	Weighted (%)	n	(%)	Weighted (%)	n	(%)	Weighted (%)
Men																		
All population (aged 25–64)	171816			150265			148465			184792			209942			199440		
All population (aged 65–94)	50251			49412			52459			49557			64506			69103		
Occupational class (EGP scheme, aged 25–64)																		
Upper non-manual workers (I+II)	39554	27.2	28.7	35046	29.6	31.3	45971	37.9	40.1	38788	35.4	37.6	41542	35.7	37.5	38124	36.0	38.1
Lower non-manual workers (III)	38274	26.3	27.9	29802	25.1	26.5	29273	24.2	25.0	27525	25.1	26.1	30340	26.1	26.9	27133	25.6	26.7
Manual workers (V+VI+VIIa)	40617	27.9	25.9	32424	27.4	25.3	25997	21.5	19.8	26211	23.9	22.1	28594	24.6	22.8	27138	25.6	23.5
Farmers (IVc+VIIb)	6550	4.5	3.3	4689	4.0	2.7	4128	3.4	2.3	4241	3.9	2.6	4344	3.7	2.7	3762	3.6	2.5
Self-employed (IVa+b)	20378	14.0	14.1	16561	14.0	14.1	15779	13.0	12.8	12766	11.7	11.6	11517	9.9	10.2	9667	9.1	9.3
Economically inactive/unknown	19976	-	-	25654	-	-	20942	-	-	19803	-	-	22722	-	-	18395	-	-
Educational level (aged 25–64)																		
Low (ISCED: 1, 2)										11558	9.3	8.5	10878	8.3	7.7	7340	6.5	5.9
Middle (ISCED: 3, 4)	N/A			N/A			N/A			68448	55.3	51.9	72362	55.3	52.0	61982	54.6	50.9
High (ISCED: 5, 6)										43800	35.4	39.6	47629	36.4	40.4	44227	38.9	43.2
Educational level (aged 65–94)																		
Low (ISCED: 1, 2)										17819	40.7	36.7	20020	36.2	32.6	17629	31.0	27.8
Middle (ISCED: 3, 4)	N/A			N/A			N/A			18951	43.3	43.3	25528	46.2	45.7	27735	48.8	47.4
High (ISCED: 5, 6)										7013	16.0	19.9	9747	17.6	21.7	11443	20.1	24.7

eTable 1. Continued

Women																		
All population (aged 25–64)	178570			157852			155990			206288			235249			221455		
All population (aged 65–94)	66228			64295			65876			62906			82011			85198		
Occupational class (EGP scheme, aged 25–64)																		
Upper non-manual workers (I+II)	19106	19.1	19.1	14592	23.5	23.7	18752	26.8	27.0	21872	24.9	24.8	24526	25.9	25.3	23566	26.5	25.9
Lower non-manual workers (III)	53089	53.2	55.8	32858	52.9	54.8	36722	52.6	54.7	48910	55.8	57.9	53716	56.7	58.7	50183	56.4	58.4
Manual workers (V+VI+VIIa)	17031	17.1	15.4	9125	14.7	12.9	8650	12.4	10.7	10341	11.8	10.7	10495	11.1	10.3	10013	11.3	10.4
Farmers (IVc+VIIb)	4889	4.9	3.9	1938	3.1	2.4	1666	2.4	1.8	2720	3.1	2.2	2512	2.7	2.0	2163	2.4	1.8
Self-employed (IVa+b)	5685	5.7	5.8	3578	5.8	6.1	4066	5.8	5.8	3882	4.4	4.4	3471	3.7	3.8	3012	3.4	3.6
Economically inactive/unknown	75274	-	-	92858	-	-	82435	-	-	51705	-	-	54422	-	-	43183	-	-
Educational level (aged 25–64)																		
Low (ISCED: 1, 2)										9332	7.1	6.5	8461	6.1	5.7	5277	4.5	4.3
Middle (ISCED: 3, 4)	N/A			N/A			N/A			79330	60.7	57.8	83039	60.3	57.6	69647	58.8	55.5
High (ISCED: 5, 6)										42110	32.2	35.8	46193	33.5	36.7	43459	36.7	40.2
Educational level (aged 65–94)																		
Low (ISCED: 1, 2)										27032	48.9	45.2	30485	43.3	39.2	26410	37.6	33.9
Middle (ISCED: 3, 4)	N/A			N/A			N/A			25190	45.5	48.0	35088	49.8	52.3	37717	53.7	55.5
High (ISCED: 5, 6)										3081	5.6	6.9	4818	6.8	8.5	6084	8.7	10.6

EGP scheme, Erikson-Goldthorpe-Portocarero scheme; ISCED, International Standard Classification of Education.

Low (ISCED: 1, 2): elementary school/junior high school graduation

Middle (ISCED: 3, 4): high school/technical professional school graduation

High (ISCED: 5, 6): 2-year college/university graduation and more

eTable 2. Definitions of occupational class

Occupational class	Occupation defined by Japanese Standard Occupational Classification	Employment status	Correspondence to the Erikson-Goldthorpe-Portocarero scheme
Upper non-manual workers	(A) Administrative and managerial workers	-	I: Higher-grade professionals, administrators and officials; managers in large industrial establishments; large proprietors
	(B) Professional and engineering workers	-	
		-	II: Lower-grade professionals, administrators and officials; higher-grade technicians; managers in small business and industrial establishments; supervisors of non-manual employees
Lower non-manual workers	(C) Clerical workers	-	III: Routine non-manual employees in administration and commerce; sales personnel; other rank-and-file service workers
	(D) Sales workers	-	
	(E) Service workers	-	
Manual workers	(H) Manufacturing process workers	-	V/VI: Lower-grade technicians; supervisors of manual workers; skilled manual workers
	(I) Transport and machine operating workers	-	
	(J) Construction and mining workers	-	
	(K) Carrying, cleaning, packaging, and related workers	-	VIIa: Semi- and unskilled manual workers (not in agriculture)
Farmers	(G) Agriculture forestry and fishery workers	-	IVc: Farmers and smallholders; self-employed fishermen<br>VIIb: Agricultural workers
Self-employed	-	Self-employed with employees, self-employed no employees	IVa: Small proprietors; artisans, etc., with employees<br>IVb: Small proprietors, artisans, etc., without employees

eTable 3. Definitions of educational level

Educational level	Educational background defined by Japanese national surveys	Correspondence to the International Standard Classification of Education (ISCED), 1997	Correspondence to the International Standard Classification of Education (ISCED), 2011
Low	Elementary school/Junior high school graduates	ISCED 1: Primary education<br>ISCED 2: Lower secondary education	ISCED 1: Primary education<br>ISCED 2: Lower secondary education
Middle	High school graduates	ISCED 3: Upper secondary education	ISCED 3: Upper secondary education
	Technical professional school graduates	ISCED 4: Post-secondary non-tertiary education	ISCED 4: Post-secondary non-tertiary education
High	2-year college graduates	ISCED 5: First stage of tertiary education	ISCED 5: Short-cycle tertiary education
	University graduates	ISCED 6: Second stage of tertiary education	ISCED 6: Bachelor’s or equivalent level
	Graduate school		ISCED 7: Master’s or equivalent level<br>ISCED 8: Doctoral or equivalent level

eTable 4. Distributions of educational level by occupational class (EGP scheme, aged 25–64)

	2010			2013			2016		
	Low (ISCED: 1, 2)	Middle (ISCED: 3, 4)	High (ISCED: 5, 6)	Low (ISCED: 1, 2)	Middle (ISCED: 3, 4)	High (ISCED: 5, 6)	Low (ISCED: 1, 2)	Middle (ISCED: 3, 4)	High (ISCED: 5, 6)
Men									
Upper non-manual workers (I+II)	2.7	40.1	57.2	2.2	39.8	58.0	1.7	38.3	60.0
Lower non-manual workers (III)	3.9	48.5	47.6	3.3	47.6	49.2	2.4	45.5	52.1
Manual workers (V+VI+VIIa)	16.2	69.9	13.9	13.8	70.9	15.3	11.2	71.2	17.6
Farmers (IVc+VIIb)	15.4	65.7	18.9	14.2	67.4	18.4	10.7	65.5	23.7
Self-employed (IVa+b)	13.4	56.0	30.6	12.9	56.6	30.4	11.5	56.8	31.8
Women									
Upper non-manual workers (I+II)	1.6	45.8	52.6	1.5	46.0	52.5	1.1	43.6	55.3
Lower non-manual workers (III)	4.4	60.6	35.0	3.8	59.7	36.5	2.9	57.9	39.2
Manual workers (V+VI+VIIa)	15.2	72.9	11.9	12.3	73.1	14.6	10.0	74.2	15.8
Farmers (IVc+VIIb)	16.8	68.9	14.3	12.3	71.7	16.0	7.6	71.7	20.7
Self-employed (IVa+b)	6.9	56.0	37.1	5.6	55.3	39.1	4.1	51.4	44.5

EGP scheme, Erikson-Goldthorpe-Portocarero scheme; ISCED, International Standard Classification of Education.

Low (ISCED: 1, 2): elementary school/junior high school graduation

Middle (ISCED: 3, 4): high school/technical professional school graduation

High (ISCED: 5, 6): 2-year college/university graduation and more

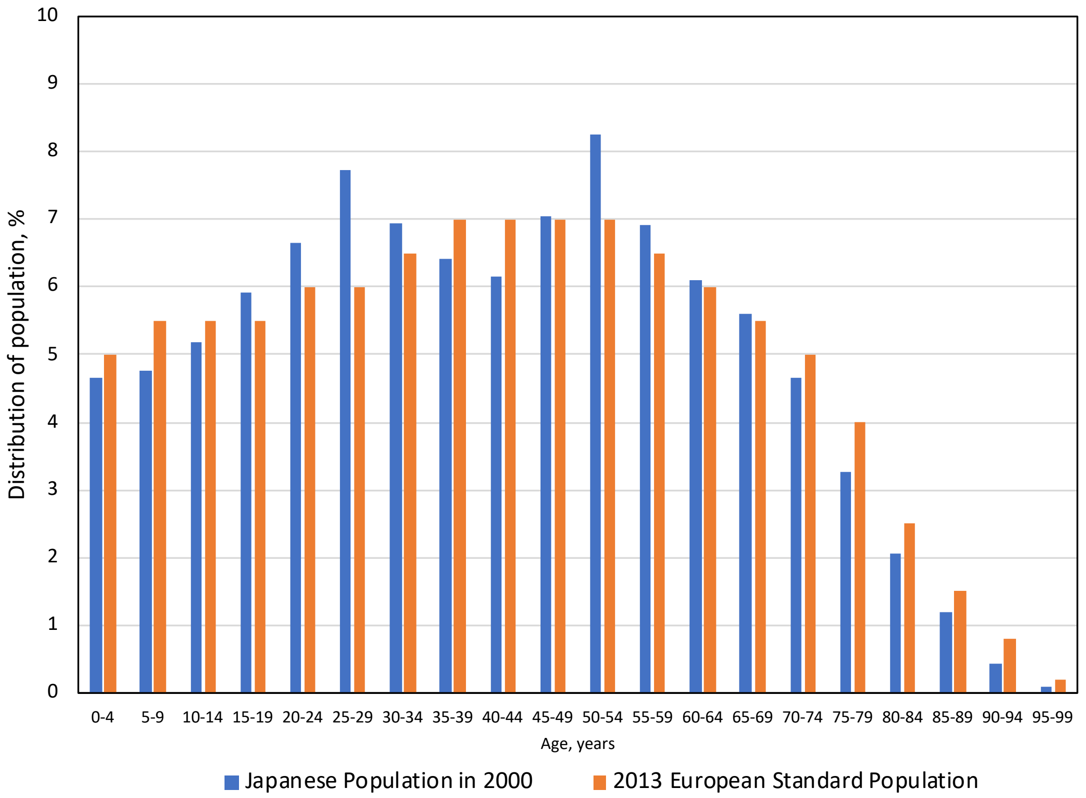

eFigure 2. Comparison of distribution between the Japanese population in 2000 and the 2013 European Standard Population

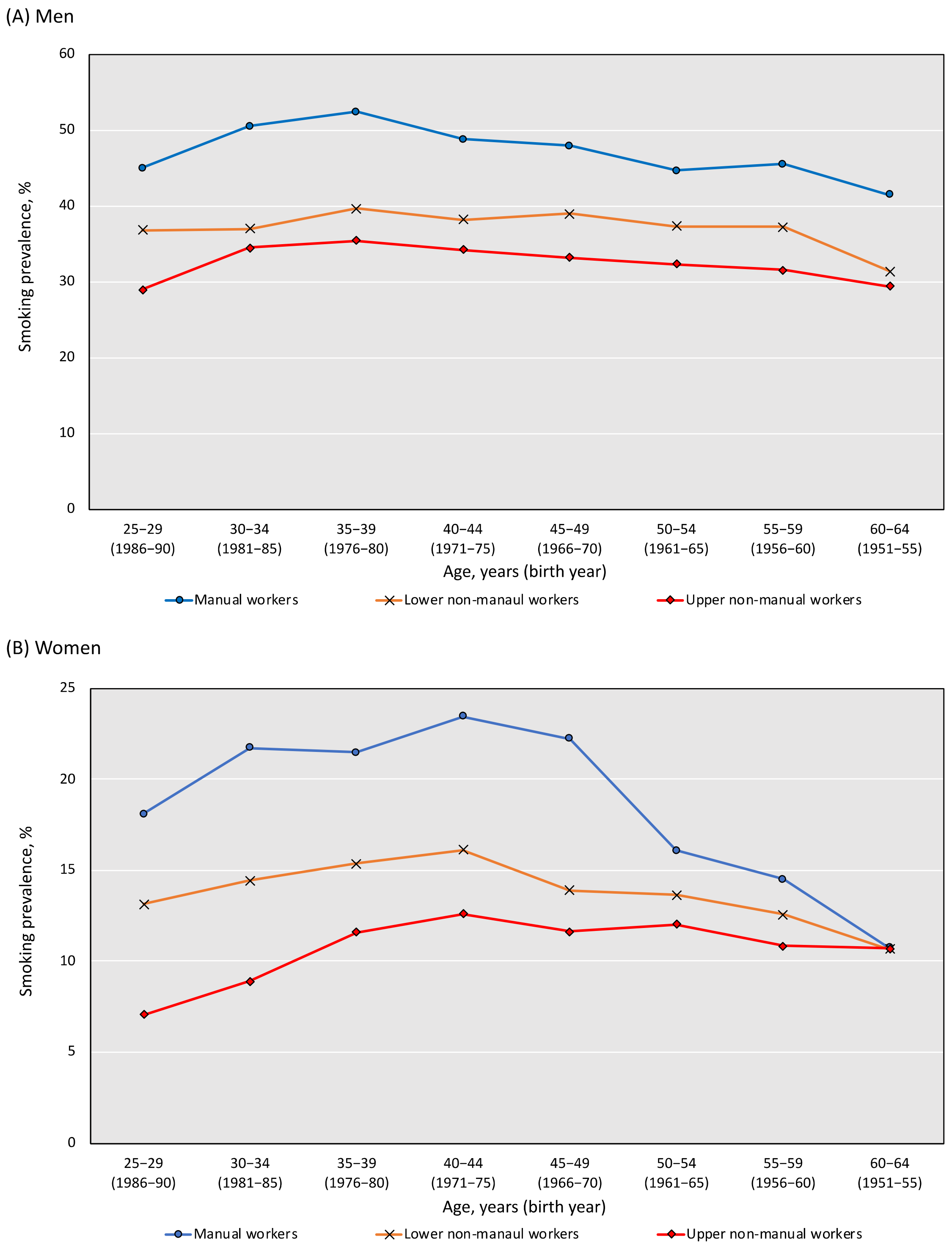

eFigure 3. Smoking prevalence by occupational class and age (birth year) in Japan in 2016

eTable 5. Trends in age-standardized smoking prevalence rates by occupational class and educational level

Survey year	2001		2004		2007		2010		2013		2016		Change		
	%	95% CI	%	95% CI	%	95% CI	%	95% CI	%	95% CI	%	95% CI	%	Percent change* %	Annual prevalence change** %
Men													(2001–2016)		
All population (aged 25—94)***	49.3	49.1 - 49.6	45.5	45.2 - 45.7	41.6	41.4 - 41.8	37.5	37.3 - 37.7	35.1	34.9 - 35.3	33.1	32.9 - 33.3	-16.3	-33.0	-1.0
All population (aged 25—64)	56.0	55.8 - 56.3	52.4	52.2 - 52.7	48.3	48.0 - 48.6	43.7	43.4 - 44.0	40.9	40.7 - 41.2	38.4	38.1 - 38.6	-17.7	-31.5	-1.1
All population (aged 65—94)	31.0	30.5 - 31.4	26.3	25.9 - 26.7	23.1	22.7 - 23.5	20.4	20.0 - 20.8	19.0	18.7 - 19.3	18.5	18.2 - 18.8	-12.4	-40.1	-0.8
Occupational class (EGP scheme, aged 25—64)													(2001–2016)		
Upper non-manual workers (I+II)	50.0	49.4 - 50.5	46.2	45.7 - 46.8	44.3	43.9 - 44.8	37.5	37.1 - 38.0	34.6	34.1 - 35.0	32.5	32.1 - 33.0	-17.4	-34.9	-1.1
Lower non-manual workers (III)	55.6	55.0 - 56.1	51.5	50.9 - 52.1	47.3	46.7 - 47.9	42.9	42.3 - 43.4	39.5	38.9 - 40.0	37.2	36.6 - 37.7	-18.4	-33.1	-1.2
Manual workers (V+VI+VIIa)	61.9	61.4 - 62.5	59.6	59.0 - 60.2	55.6	54.9 - 56.2	53.3	52.7 - 53.9	50.7	50.1 - 51.3	47.1	46.5 - 47.7	-14.8	-23.9	-0.9
Farmers (IVc+VIIb)	58.1	56.4 - 59.7	55.0	53.1 - 57.0	53.8	51.6 - 55.9	49.4	47.4 - 51.5	48.3	46.3 - 50.3	45.8	43.7 - 47.8	-12.3	-21.2	-0.8
Self-employed (IVa+b)	61.2	60.4 - 62.0	57.6	56.7 - 58.5	53.1	52.1 - 54.0	48.1	47.1 - 49.2	46.5	45.4 - 47.7	45.3	44.0 - 46.5	-16.0	-26.0	-1.0
Economically inactive/unknown	54.7	53.9 - 55.5	51.7	0.0 - 0.0	48.0	47.2 - 48.8	44.4	43.6 - 45.2	41.7	41.0 - 42.4	38.3	37.5 - 39.0	-16.5	-30.1	-1.0
Educational level (aged 25—64)													(2010–2016)		
Low (ISCED: 1, 2)							60.0	58.9 - 61.0	58.4	57.4 - 59.5	57.8	56.6 - 59.0	-2.2	-3.6	-0.4
Middle (ISCED: 3, 4)	N/A		N/A		N/A		49.2	48.8 - 49.6	45.9	45.5 - 46.3	43.9	43.5 - 44.4	-5.3	-10.7	-0.9
High (ISCED: 5, 6)							32.9	32.5 - 33.4	30.1	29.7 - 30.5	27.8	27.4 - 28.2	-5.2	-15.7	-0.9
Educational level (aged 65—94)													(2010–2016)		
Low (ISCED: 1, 2)							22.8	22.1 - 23.5	22.0	21.3 - 22.6	21.6	20.9 - 22.2	-1.2	-5.3	-0.2
Middle (ISCED: 3, 4)	N/A		N/A		N/A		20.5	19.9 - 21.1	18.6	18.1 - 19.1	18.5	18.1 - 19.0	-2.0	-9.8	-0.3
High (ISCED: 5, 6)							15.2	14.4 - 16.0	14.0	13.4 - 14.7	14.1	13.5 - 14.7	-1.1	-7.1	-0.2

eTable 5. Continued

Women													(2001–2016)		
All population (aged 25–94)***	14.1	14.0 - 14.3	13.8	13.7 - 14.0	13.6	13.4 - 13.7	12.1	12.0 - 12.2	11.7	11.6 - 11.9	10.7	10.6 - 10.9	-3.4	-24.0	-0.2
All population (aged 25–64)	17.0	16.8 - 17.2	16.9	16.7 - 17.1	16.6	16.5 - 16.8	14.9	14.7 - 15.1	14.3	14.2 - 14.5	13.0	12.8 - 13.1	-4.1	-23.9	-0.3
All population (aged 65–94)	6.1	5.8 - 6.3	5.4	5.2 - 5.6	5.1	4.9 - 5.2	4.5	4.3 - 4.6	4.6	4.4 - 4.7	4.5	4.4 - 4.7	-1.5	-24.8	-0.1
Occupational class (EGP scheme, aged 25–64)													(2001–2016)		
Upper non-manual workers (I+II)	14.7	14.1 - 15.3	14.3	13.7 - 15.0	14.8	14.2 - 15.4	12.2	11.8 - 12.7	11.4	11.0 - 11.8	10.8	10.4 - 11.2	-4.0	-26.9	-0.2
Lower non-manual workers (III)	18.6	18.2 - 19.0	19.6	19.1 - 20.1	19.3	18.8 - 19.7	16.1	15.8 - 16.5	15.6	15.3 - 15.9	13.8	13.5 - 14.1	-4.8	-25.9	-0.3
Manual workers (V+VI+VIIa)	18.8	18.1 - 19.5	20.4	19.4 - 21.3	23.1	22.0 - 24.1	19.6	18.7 - 20.5	20.7	19.8 - 21.6	18.7	17.9 - 19.6	-0.1	-0.5	0.0
Farmers (IVc+VIIb)	14.2	12.7 - 15.7	9.8	7.5 - 12.1	15.4	11.9 - 18.8	10.4	8.3 - 12.4	13.2	10.9 - 15.4	11.5	9.2 - 13.7	-2.8	-19.5	-0.2
Self-employed (IVa+b)	27.2	25.8 - 28.7	28.4	26.7 - 30.1	25.5	23.9 - 27.0	20.8	19.3 - 22.2	19.3	17.7 - 20.9	16.7	15.1 - 18.3	-10.5	-38.6	-0.7
Economically inactive/unknown	15.9	15.6 - 16.2	15.9	15.7 - 16.2	15.3	15.1 - 15.6	14.0	13.7 - 14.3	13.2	12.9 - 13.5	12.2	11.9 - 12.5	-3.8	-23.6	-0.2
Educational level (aged 25–64)													(2010–2016)		
Low (ISCED: 1, 2)							37.9	36.5 - 39.2	37.8	36.5 - 39.1	34.7	33.3 - 36.1	-3.1	-8.3	-0.5
Middle (ISCED: 3, 4)	N/A		N/A		N/A		17.5	17.2 - 17.8	17.3	17.0 - 17.5	15.9	15.6 - 16.2	-1.6	-9.0	-0.3
High (ISCED: 5, 6)							7.4	7.2 - 7.7	6.5	6.3 - 6.8	5.6	5.4 - 5.8	-1.8	-24.9	-0.3
Educational level (aged 65–94)													(2010–2016)		
Low (ISCED: 1, 2)							5.4	5.1 - 5.8	5.7	5.4 - 6.0	5.9	5.6 - 6.3	0.5	9.2	0.1
Middle (ISCED: 3, 4)	N/A		N/A		N/A		3.8	3.5 - 4.0	4.0	3.8 - 4.2	4.0	3.8 - 4.2	0.2	5.3	0.0
High (ISCED: 5, 6)							2.7	2.1 - 3.2	3.2	2.7 - 3.7	2.9	2.5 - 3.2	0.2	7.0	0.0

*Difference percentages expressed as percentages of 2001 (for occupational class) or 2010 (for educational level).

**Estimated from prevalence of two points between 2001-2016 (for occupational class) or 2010-2016 (for educational level).

***Survey participants aged 20-24 years old were excluded because this age band includes university and other higher education students.

CI, confidence Interval; EGP scheme, Erikson-Goldthorpe-Portocarero scheme; ISCED, International Standard Classification of Education.

Low (ISCED: 1, 2): elementary school/junior high school graduation

Middle (ISCED: 3, 4): high school/technical professional school graduation

High (ISCED: 5, 6): 2-year college/university graduation and more
